# Use of vaccines and factors associated with their uptake variability in dogs, cats and rabbits attending a large sentinel network of veterinary practices across Great Britain

**DOI:** 10.1017/S0950268818000754

**Published:** 2018-04-11

**Authors:** F. Sánchez-Vizcaíno, A. Muniesa, D. A. Singleton, P. H. Jones, P. J. Noble, R. M. Gaskell, S. Dawson, A. D. Radford

**Affiliations:** 1 NIHR Health Protection Research Unit in Emerging and Zoonotic Infections, University of Liverpool, UK; 2 Institute of Infection and Global Health, Leahurst Campus, Chester High Road, Neston, S. Wirral, CH64 7TE, UK; 3 Department of Animal Pathology, Faculty of Veterinary Sciences, Instituto Agroalimentario de Aragón (IA2) (Universidad de Zaragoza-CITA), c/ Miguel Servet 177, 50013 Zaragoza, Spain; 4 Veterinary Institute, University of Liverpool, Leahurst Campus, Chester High Road, Neston, S. Wirral, CH64 7TE, UK

**Keywords:** Vaccination (immunization), vaccines, veterinary pathogens, veterinary virology

## Abstract

Vaccination remains a mainstay of companion animal population health. However, how vaccine use at a population level complies with existing guidelines is unknown. Here we use electronic health records to describe vaccination in dogs, cats and rabbits attending a large sentinel network of UK veterinary practices. In total, 77.9% (95% CI: 77.6–78.1) of animals had recorded vaccinations. The percentage of animals with recorded vaccinations was higher in dogs, neutered animals, in insured dogs and cats and in purebred dogs. Vaccination rates varied in different regions of Great Britain in all species. Dogs and cats belonging to owners living in less deprived areas of England and Scotland were more likely to be recorded as vaccinated. In the vaccinated population, cats received more core vaccines per year of life (0.86) than dogs (0.75), with feline leukaemia vaccines almost as frequent as core vaccines. In dogs, leptospira vaccines were more frequent than core vaccines. This descriptive study suggests a substantial proportion of animals are not benefiting from vaccine protection. For the first time, we identify potential factors associated with variations in recorded vaccination frequency, providing a critical baseline against which to monitor future changes in companion animal vaccination and evidence to inform future targeted health interventions.

## Introduction

Vaccination is one of the primary drivers to improve population health, reducing morbidity and mortality and in some cases, leading to disease eradication. Vaccination has been part of companion animal health since the first introduction of rabies vaccines [1]. In most cases they have been developed to meet pet health markets; notable exceptions include rabies, which is also important for human health and vaccines to protect rabbits, important food animals in many countries.

Vaccines are a prescription only medicine, their use in Europe overseen by competent authorities in each country and increasingly, by the European Medicines Agency. For a veterinary vaccine to be authorised in Europe their safety, efficacy and duration of immunity have to be demonstrated under carefully controlled conditions [2]. Financial and welfare constraints of these experiments have meant most vaccines were initially authorised with a 1 year duration of immunity (DOI), leading to the evolution of annual pet vaccinations. However, several factors, most notably rare adverse events post-vaccination [3] and a growing realisation that some vaccines can have durations of immunity much beyond a single year [4–6] are leading to a gradual challenge to this practice.

These sometimes conflicting pressures, coupled with an absence of precise data, has led to the establishment of guideline groups which seek to use all current evidence, from controlled trials to expert opinion, to balance the risks and benefits of vaccination and to advise practitioners how often to vaccinate individual animals [7–9]. Such guidelines consistently point to a more tailored approach to vaccination with each animal's vaccination schedule based on their local risk. Each guideline group has defined ‘core’ vaccines; the set of antigens that all animals of a given species should benefit from. For dogs, these include canine parvovirus (CPV), canine distemper virus (CDV) and canine adenovirus (CAV) and in some countries, rabies virus [10]. For cats, core vaccines include feline calicivirus (FCV), feline herpesvirus (FeHV-1) and feline panleucopaenia (FPLV) [9, 10]. Other ‘non-core’ vaccine antigens are recommended to be used in a more tailored way based on each individual's risk. Most guideline groups agree on the need for core vaccines at 9 and 12 weeks, with a booster 1 year later, followed by repeat vaccines every 1–3 years, depending on the antigen and the level of perceived risk [8–10]. Uncertainty over the period that maternally derived antibodies interfere with vaccination has led some to also suggest puppy and kitten vaccines both earlier and later [10]. Vaccination guidelines have now been available for over 10 years. However, to what extent these guideline messages are being applied in veterinary practice is not known.

In the UK, which has a highly developed pet industry, it is estimated that 77% of owned dogs and 86.4% of owned cats are registered with a veterinary practice [11, 12]. Here we use electronic health records to describe vaccination schedules and how these comply with existing guidelines as well as to identify potential factors associated with variations in recorded vaccination frequency in cats, dogs and rabbits attending a large sentinel network of veterinary practices across Great Britain.

## Methods

### Data collection

Electronic health records (EHRs) were collected in near real-time through the Small Animal Veterinary Surveillance Network (SAVSNET) from volunteer veterinary practices across Great Britain; a full description of the data collection protocol has been described elsewhere [13, 14]. Briefly veterinary practices using practice management software previously made compatible with SAVSNET participation and data exchange were recruited based on convenience. In each participating practice, data are collected from each booked consultation as long as the owner of the pet does not first opt out. This cross-sectional study uses the last recorded consultation for each dog, cat and rabbit attending SAVSNET veterinary practices between 22 November 2013 and 1 October 2015 along with its full recorded vaccination history (‘Total Population’). Each EHR contained identifiers for practice, premises and animal, the animal's species, breed, sex and dates of birth, neutering and insurance, full owner's postcode, recorded vaccination dates and vaccine codes (a text string defined by individual practices e.g. ‘VaccinationDogDhppi/L’).

### Data management

The text-based data for breed were cleaned to deal with misspellings or non-standard terms by mapping to standard terms as previously [14]. A list of 2084 unique vaccine codes was identified and manually categorised by a domain expert (ADR) to standard vaccine names (e.g. ‘Vaccination Dhppi/L’ and ‘Dog1^st^VaccDHPPi + L’ were categorised as ‘DHPPiL’). Vaccine codes where the antigen could not be identified (e.g. ‘booster’) were classified as ‘vaccine unknown antigen’.

The pet owner's postcode was used to define a geographic location (i.e. country and region – Nomenclature of Units for Territorial Statistics (NUTS) level 1) and link to Indexes of Multiple Deprivation (IMD) as described elsewhere [14]. Ranks of IMD for England, Wales and Scotland were independently categorised based on quintile cut-off scores with category 1 being least deprived.

In this study, animals who had ever been recorded with a vaccination event were considered as vaccinated. The neutering and insurance status was positively recorded if the animal had ever been recorded as neutered and/or insured between 22 November 2013 and 1 October 2015. Dogs and cats with an age or age of vaccination outside the range 0–26 years were excluded; for rabbits, the age range was 0–16 years. Some practices had inevitably not been using compliant versions of practice software for as long as some of their patients have lived; in order to maximise the chances that full vaccination histories could be captured, animals were only included in this study if their date of birth was after the first recorded date of any vaccination for any animal attending the same practice. Forty-four animals for which a vaccination date was not accurately recorded for at least one vaccination event were also removed. The remaining dataset constituted the ‘Study Population’. A second ‘Full Antigen History’ dataset was derived by also excluding those animals whose recorded vaccination histories contained any individual vaccines containing unknown antigens (for example ‘Booster’ or ‘puppy vaccination’). Core vaccines were defined in dogs as CPV, CDV and CAV; and in cats as FeHV-1-1, FCV and FPV [10]. To the authors’ knowledge, core vaccines are not defined for rabbits.

### Statistical analysis

Proportions and confidence intervals (95%) were calculated to adjust for clustering within premises using the ‘varbin’ function (binomial method) implemented in R ‘aods3’ [15].

Paired *t*-tests were used for a matched-pairs premise-level sample to investigate at species level whether the mean recorded percentage of vaccinated animals was significantly different (i) in purebred compared with crossbred animals; (ii) in neutered compared with entire animals; and (iii) in insured compared with non-insured animals. Prior to analysis, the normality of paired differences was determined by visual examination of quantile–quantile plots and confirmed by a Shapiro–Wilk normality test (*P* > 0.05) (results not shown).

A Spearman's rank-order correlation coefficient was used for testing association between recorded percentage of vaccinated dogs and cats at the premise-level. This non-parametric method was preferred because the two variables assessed were non-normal distributed with outliers (results not shown).

Mixed-effects binary logistic regression models were used to assess the strength of association between the fixed effect age and several outcome variables such as the probability of dogs, cats or rabbits being recorded as vaccinated. Age was centred to make model interpretation easier.

Further, mixed effects binary logistic regression models were used to assess the strength of association between the fixed effect IMD and several outcome variables such as the probability of dogs, cats or rabbits being recorded as vaccinated. Separate models were undertaken for animals living in England, Scotland and Wales as IMD measures between these countries are not directly comparable. For each fitted model, a likelihood ratio test (LRT) was conducted to evaluate the statistical significance of the fixed effect IMD (i.e. considering all IMD categories together). The strength of association between IMD and an outcome variable was shown only for models where the fixed effect IMD was statistically significant.

On each dataset used for fitting the mixed effects models described, a prior LRT was performed to evaluate the statistical significance of each random effect *vs.* the other (i.e. ‘practice'Vvs.’premise’) and *vs.* both random effects included. When both random effects included did not improve the fit of the model, the individual random effect that better fitted the model was selected to follow the principle of parsimony.

The models were fitted using the Laplace approximation implemented in the R package ‘lme4’.

Statistical significance was defined as *P* < 0.05. All statistical analyses were carried out using R language (v3.3.2) [16].

## Results

### Total population

A total of 825 375 EHRs (from 311 178 unique animals) were collected from 136 practices (313 premises). For each unique animal, the data included the last recorded consultation date between 22 November 2013 and 1 October 2015 along with their recorded collated vaccination history, which ranged between 30 October 1997 and 1 October 2015. The data consisted of 592 544 dog EHRs (208 716 individual dogs), 222 092 cat EHRs (95 809 cats) and 10 739 rabbit EHRs (6653 rabbits) from 136 practices (313 premises), 136 practices (310 premises) and 130 practices (264 premises) respectively.

### Study population

After the data managing process, the Study Population consisted of 330 904 EHRs, which represented 116 745 animals from 101 practices (244 premises). This dataset included 237 949 dog EHRs (77 980 unique dogs), 86 728 cat EHRs (34 930 cats) and 6227 rabbit EHRs (3835 rabbits) from 100 practices (241 premises), 101 practices (238 premises) and 96 practices (186 premises) respectively. Vaccination dates ranged between 24 March 1998 and 1 October 2015.

With regard to regression modelling, for each dataset used to fit a mixed effects model, the results of a LRT comparing models incorporating only ‘practice’ or ‘premise’ or both as random effects are summarised in Supplementary Table 1.

Of the animals in the Study Population, 77.9% (95% CI: 77.6–78.1%) had recorded vaccinations including 81.5% of dogs (81.2–81.7%), 73.1% of cats (72.6–73.5%) and 48.4% of rabbits (46.8-49.9%); compared with dogs, cats and rabbits were 1.4 and 2.8 times more likely to have no recorded vaccine. Recorded vaccination decreased slightly to 73.7% (73.3–74.2) when only animals <1 year of age were included; dog 79.0% (78.5–79.5), cat 66.1% (65.2–66.9) and rabbit 42.8% (40.2–45.5). A positive relationship was found between recorded vaccination and age, with 2.2%, 3.7% and 13.3% increase in the odds of being vaccinated for a one-unit increase in years of age in dogs, cats and rabbits, respectively (*P* < 0.001 all species) (Table 1).

**Table 1.** Parameter estimates from three mixed-effects logistic regression models, assessing the association between being recorded as vaccinated and age for dogs, cats and rabbits attending a network of veterinary practices across Great Britain

Species	Random effects		Fixed effects				
	Parameter	Variance	Parameter	Beta	s.e.	OR (95% CI)	*P* value
Dog	Practice	0.272	Intercept	1.587	0.058	4.887 (4.358–5.481)	<0.001
	Premise	0.040	Age centred^a^	0.021	0.004	1.022 (1.013–1.030)	<0.001
Cat	Practice	0.154	Intercept	0.979	0.052	2.662 (2.406–2.946)	<0.001
	Premise	0.088	Age centred^a^	0.036	0.005	1.037 (1.027–1.047)	<0.001
Rabbit	Practice	1.045	Intercept	−0.129	0.133	0.879 (0.677–1.142)	0.334
	Premise	0.140	Age centred^a^	0.125	0.021	1.133 (1.088–1.179)	<0.001

SE, standard error; OR, odds ratio; CI, confidence interval.

a Age centred: mean age was 2.8 years in dogs, 3.0 years in cats and 2.3 years in rabbits.

**Fig. 1.**
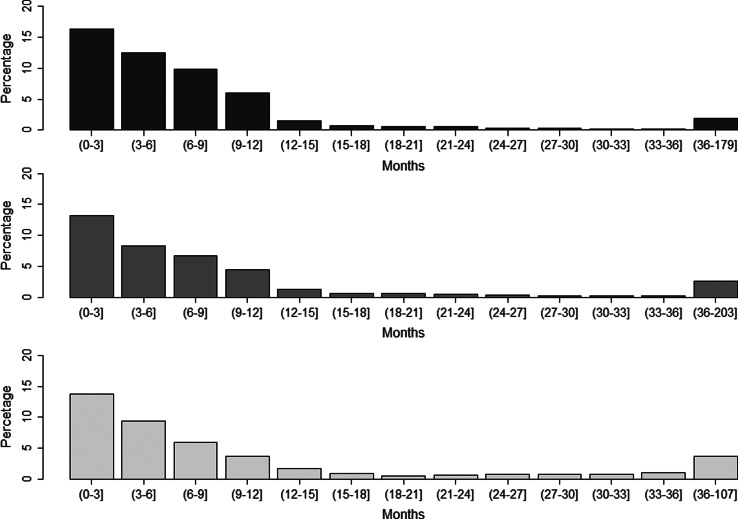
Proportion of (a) dogs, (b) cats and (c) rabbits with at least one recorded vaccine date by time from the last recorded vaccine in animals attending a network of veterinary practices across Great Britain. For age in months, the first number is included in the interval (indicated by curly bracket), whereas the second number is excluded (indicated by square bracket).

The average number of recorded vaccines per year of life was 1.04, 0.74 and 0.48 for dogs, cats and rabbits, respectively. For those animals with at least one recorded vaccine date, the percentages whose last recorded vaccine was more than 1 year or more than 3 years before the date of the last recorded consultation was 6.5% and 1.9% for dogs, respectively, 6.7% and 2.6% for cats and 10.9% and 3.7% for rabbits (Fig. 1).

Mapped breeds were available for 96.6% of dogs, cats and rabbits in the Study Population. Where a mapped breed was available, 87.5% of dogs, 14.6% of cats and 98.3% of rabbits were recorded as purebred. The recorded percentage of vaccinated animals was significantly higher in purebred dogs (81.9%, 95% CI: 81.6–82.2) than crossbred dogs (79.7%, 78.9–80.5) (paired *t* test, *P* = 0.008), but was not significantly different between purebred (73.8%, 72.5–75.02) and crossbred cats (74.1%, 73.6–74.7) (*P* = 0.09). Rabbits were excluded from these analyses because crossbred rabbits were underrepresented in a large number of premises.

In the Study Population, 44.9% of dogs, 66.6% of cats and 40.6% of rabbits were neutered. Vaccination was significantly more common in neutered dogs (84.7%, 95% CI: 84.4–85.1), cats (77.7%, 77.2–78.2) and rabbits (57.2%, 54.7–59.6) than in entire dogs (78.7%, 78.3–79.1), cats (63.6%, 62.7–64.5) and rabbits (41.6, 39.6–43.7) (paired *t* test, *P* < 0.001 all species).

The recorded insurance frequency of dogs, cats and rabbits was 18.9%, 10.4% and 2.5%, respectively. Vaccination was significantly more commonly recorded in insured dogs (89.1%, 95% CI: 88.6–89.6) and cats (86.7%, 85.6–87.8) than in uninsured dogs (79.6%, 79.2–79.9) and cats (71.5%, 71.0–72.0) (paired *t* test, *P* < 0.001 in both species). Rabbits were excluded from these results because insured rabbits were underrepresented in large numbers of premises.

**Fig. 2.**
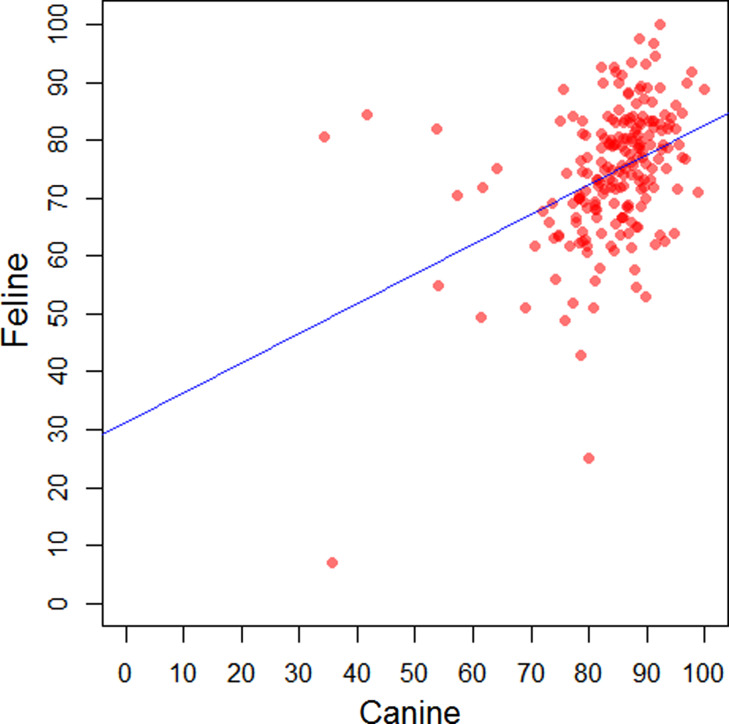
Comparison of the recorded percentage of vaccinated dogs and the recorded percentage of vaccinated cats by premises in a network of veterinary practices across Great Britain.

The regions (NUTS 1) of GB with lowest and highest levels of recorded vaccination were respectively, Scotland (75.4%, 95% CI: 74.3–76.4) and East of England (86.1%, 85.0–87.1) in dogs; Wales (56.4, 54.4–58.4) and East of England (77.9, 76.1–79.6) in cats; and North West (34.7, 31.4–38.1) and South East (65.4, 61.7–69.0) in rabbits (Supplementary Table 2). A positive moderate correlation was found between the recorded percentage of vaccinated dogs and the recorded percentage of vaccinated cats at the premises-level (*r*_*s*_ = 0.42, *P* < 0.001) (Fig. 2).

The distribution of animals by species, country and IMD category is shown in Supplementary Table 3. A significant relationship was found between being recorded as vaccinated and the predicted IMD for dogs and cats attending SAVSNET practices whose owners were living in England (dogs: *χ*^2^_df=4_ = 162, *P* < 0.001; cats: *χ*^2^_df=4_ = 174.02, *P* < 0.001) or Scotland (dogs: *χ*^2^_df=4_ = 14.26, *P* = 0.006; cats: *χ*^2^_df=4_ = 36.05, *P* < 0.001) and for rabbits whose owners were living in England (*χ*^2^_df=4_ = 28.16, *P* < 0.001). For dogs and cats, the odds of being recorded as vaccinated were significantly higher if their owners lived in less deprived areas of England and Scotland rather than the most deprived areas (dogs: *P* < 0.05 for IMD categories 1–4 in England and for IMD categories 1–3 in Scotland; cats: *P* < 0.05 for IMD categories 1–4 in England and for IMD categories 1–2 in Scotland) (Table 2). This relationship was not significant for animals whose owners lived in Wales (dogs: *χ*^2^_df=4_ = 6.48, *P* = 0.17; cats: *χ*^2^_df=4_ = 8.91, *P* = 0.06).

**Table 2.** Parameter estimates from five mixed-effects logistic regression models, assessing the association between being recorded as vaccinated and the Index of Multiple Deprivation (IMD) for dogs, cats and rabbits attending a network of veterinary practices across Great Britain whose owners were living in England (for all species) or in Scotland (only dogs and cats)

Country	IMD	Dog vaccinated: yes		Cat vaccinated: yes		Rabbit vaccinated: yes	
		Odds ratio (95% CI)		Odds ratio (95% CI)		Odds ratio (95% CI)	
England	5	Reference	–	Reference	–	Reference	–
	4	1.32	(1.20–1.44)***	1.40	(1.25–1.56)***	0.62	(0.41–0.93)*
	3	1.33	(1.21–1.45)***	1.61	(1.44–1.80)***	1.26	(0.87–1.83)
	2	1.62	(1.49–1.78)***	1.80	(1.61–2.02)***	1.37	(0.95–1.98)
	1	1.71	(1.56–1.88)***	2.15	(1.91–2.42)***	1.16	(0.80–1.68)
Scotland	5	Reference	–	Reference	–		
	4	1.18	(0.89–1.55)	1.17	(0.79–1.74)		
	3	1.41	(1.08–1.83)*	1.44	(0.99–2.11)		
	2	1.50	(1.15–1.95)**	2.40	(1.63–3.53)***		
	1	1.55	(1.18–2.04)**	2.32	(1.55–3.47)***		

***P < 0.001; **P < 0.01; and *P < 0.05. CI, confidence interval.

### Animals with a full vaccine antigen history

This ‘Full Antigen History’ dataset consisted of 156 824 EHRs (50 325 unique animals) from 99 practices (233 premises). The data included 93 127 dog EHRs (29 274 unique dogs) from 94 practices (210 premises), 59 626 cat EHRs (19 233 unique cats) from 96 practices (221 premises) and 4071 rabbit EHRs (1818 unique rabbits) from 88 practices (157 premises).

The percentage of animals receiving each antigen and the average number of recorded vaccines per year of life is summarised in Table 3. In dogs, *Leptospira* vaccines and core vaccines were recorded in most animals (95.5% (95% CI: 95.3–95.8) and 91.5% (91.2–91.8) respectively). Parainfluenza (70.2%, 69.6–70.7) and *Bordetella* (36.7%, 36.1–37.2) vaccines were also quite frequently used, whereas rabies vaccines were rarely recorded (3.3%; 3.1–3.5). In cats, core vaccines and feline leukaemia virus (FeLV) vaccines were recorded in most animals (96.6% (96.3–96.8) and 88.7% (88.3–89.2) respectively), whereas other antigens such as *Bordetella* (0.01%, 0.003–0.04), *Chlamydophila* (0.9%, 0.8–1.0) and rabies (0.7%, 0.6–0.8) were only recorded infrequently. Myxomatosis and rabbit haemorrhagic disease virus vaccines were recorded for most rabbits in this population (99.2% (98.7–99.5) and 97.0% (96.1–97.7), respectively).

**Table 3.** Recorded vaccination percentages for each pathogen and average number of recorded vaccines per year of life in dogs (*n* = 29 274) cats (*n* = 19 233) and rabbits (*n* = 1818) attending a network of veterinary practices across Great Britain

Species	Pathogen	Mean percentage of animals with a recorded vaccination (95% CI)	Average number of recorded vaccines per year of life
Dog	*Leptospira*	95.5 (95.3–95.8)	0.93
	Canine parvovirus	91.9 (91.6–92.2)	0.69
	Canine distemper virus	91.5 (91.2–91.8)	0.69
	Canine hepatitis	91.5 (91.2–91.8)	0.69
	Core	91.5 (91.2–91.8)	0.69
	Parainfluenza	70.2 (69.6–70.7)	0.74
	*Bordetella*	36.7 (36.1–37.2)	0.54
	Rabies	3.3 (3.1–3.5)	0.35
	*Leishmania*	0.01 (0.005–0.03)	1.04
Cat	Feline calicivirus	98.4 (98.2–98.5)	0.86
	Feline herpesvirus	98.4 (98.2–98.5)	0.86
	Feline panleukopenia virus	96.6 (96.3–96.8)	0.85
	Core	96.6 (96.3–96.8)	0.85
	Feline leukemia virus	88.7 (88.3–89.2)	0.84
	*Clamydophila*	0.9 (0.8–1.0)	0.9
	Rabies	0.7 (0.6–0.8)	0.27
	*Bordetella*	0.01 (0.003–0.04)	0.24
Rabbit	Myxomatosis	99.2 (98.7–99.5)	0.88
	Rabit haemorragic disease virus	97.0 (96.1–97.7)	0.82

CI, confidence interval.

**Fig. 3.**
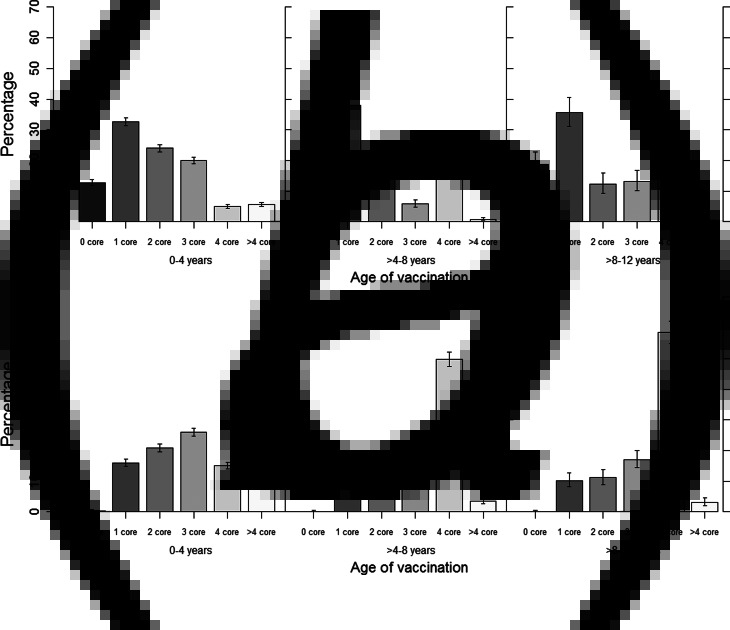
Percentage of each number of core vaccines recorded as given over certain periods of an animal's life (i.e. the first 4 years, 4–8 years, and 8–12 years) in dogs (a) and cats (b) attending a network of veterinary practices across Great Britain.

**Fig. 4.**
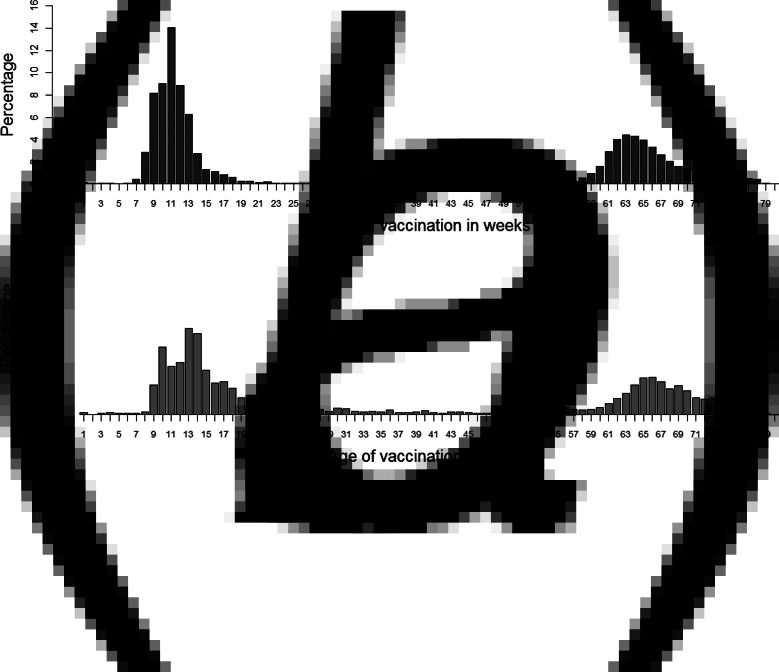
The timing of recorded administration of core vaccines over the first 18 months of life in dogs (a) and cats (b) attending a network of veterinary practices across Great Britain.

Cats received on average more vaccines containing core antigens per year of life (0.85) than dogs (0.69). In cats recorded as receiving at least one vaccine containing core antigens, 10.2%, 8.1% and 3.4% of animals were >12 months, >14months and >36 months from their last core vaccine. For dogs recorded with at least one vaccine containing core antigens, 31.5%, 23.6% and 3.0% were >12 months, >14months and >36 months from their last core vaccine. Animals that were within 12 months of their last core vaccine include animals whose last SAVSNET consultation was for a core vaccine and those within 12 months of their last vaccine and were not further analysed. Dogs were most likely to receive a single vaccine containing core antigens in any given 4-year period (Fig. 3). In contrast, cats were most likely to receive three vaccines containing core antigens in the first 4 years of life and four vaccines containing core antigens between years 4–8 and years 8–12 (Fig. 3). The timing of recorded administration of these core antigen-containing vaccines over the first 18 months of life is shown in Figure 4, highlighting recorded puppy and kitten vaccines in the first months of life (the primary vaccination course) and the recorded booster vaccines 1 year later. The primary vaccination course was generally administered over a shorter time period in dogs with 82.7% of core vaccines given between 8 and 14 weeks of age, compared with 53.3% in cats. In dogs and cats, only 6.4 and 2.3% of the primary core vaccines, respectively, were recorded at <9 weeks. Over one quarter of animals lacked a recorded booster vaccination, with 26.5% of dogs and 30.5% of cats that were over 18 months of age and that received at least one core vaccine when they were <6 months of age having no record of a core vaccine between 12 and 18 months of age.

## Discussion

Despite its importance [17], accurate data on pet animal vaccination remains elusive [10], partly because the pet animal sector lacks national oversite of population health. Here we identify for the first time the size of the vaccinated population in a sentinel network of veterinary practices and describe lower levels of vaccine uptake in some animal groups.

Over 20% of animals in this study population had no recorded vaccination. These proportions were higher in animals <1 year of age, reducing only slightly as animals aged, such that even older age groups contained a significant proportion of unvaccinated animals. These levels of non-vaccination were higher than in a pet owner survey suggesting 12% of dogs, 18% of cats and 37% of rabbits had never been vaccinated [18]. Such differences may be explainable by population or methodological differences between the two surveys, such as owner recall bias. However, the relative vaccination of each species was similar; compared with dogs, cats and rabbits were 1.4 and 2.8 times less likely to have a recorded vaccine, respectively, in our study and 1.5 and 3.1 times less likely to have a recorded vaccine in the owner study [18]. These unvaccinated animals help vaccine–preventable pathogens persist in the population.

Factors positively associated with having a recorded vaccine history included being pedigree (dogs), being neutered (dogs, cats, rabbits), being insured (cats, dogs) and living in less deprived areas of England (dogs, cats, rabbits) and Scotland (dogs, cats). The lack of such an observation for Wales may either suggest insufficient data or other factors not captured in this study are involved. Lower vaccine uptake in areas of higher socioeconomic deprivation is also observed for human vaccinations even though vaccines are often free in the studied populations [19, 20]. However, a web-based survey of owners of vaccinated and unvaccinated cats found no significant association with household income [21]. Further work is needed to explore which individual components of socioeconomic deprivation as reported here, including predictions of household income, educational attainment and employment, impact most on recorded vaccine uptake. Results also suggested the practice and premise a dog or cat attended could be associated with the vaccination uptake in individual animals although further studies to understand their role are needed.

One of the challenges of using EHRs is the variable depth of data recorded by veterinary practitioners. In some cases, vaccination details are limited to descriptive terms like ‘vaccination’ and ‘booster’. Here we defined a cohort of animals for which full antigen history was recorded. In this vaccinated population, the number of vaccine events (any antigen) recorded per year of life was 1.27, 0.98 and 0.97 for dogs, cats and rabbits, respectively, suggesting overall dogs receive more vaccine products than other species.

Most established vaccine guideline groups have defined a ‘core’ set of vaccine antigens that every animal should benefit from [8–10]. Interestingly, and in contrast to overall vaccines, cats received more core vaccines per year of life (0.85) than dogs (0.69). This was evident in all age groups with dogs most likely to receive one core vaccine in each 4-year period of life, whereas cats were more likely to receive three (years 0–4) or four (years 5–8 and 9–12), suggesting vaccine guidelines and information around prolonged DOI may have penetrated the canine sector in this population more than cats. When veterinary vaccines are authorised in the UK, the European Pharmacopoeia requires controlled challenge experiments to support DOI claims [2]. Practical issues around cost and animal welfare meant most vaccines were initially authorised with a 1 year, effectively minimum, DOI. In the USA, most vaccines did not need to demonstrate DOI but were generally authorised labelled ‘annual revaccination necessary’ [22]. In 2001, new guidance encouraged companies to calculate actual DOIs. For dog vaccination, a clear consensus developed over several years supporting prolonged DOIs and booster vaccination every three years both in guideline groups [10, 23], and in the literature [5, 24, 25] and many currently authorised canine core vaccines include a 3-year DOI. In cats, however, the picture is more complex. Most accept the highly immunogenic feline panleukopaenia virus requires 3-yearly adult vaccination [8–10]. However, FCV genetic [26] and antigenic [27–29] variation, and relatively low antibody responses to FeHV-1 [4, 30], has meant that feline core vaccines with 3-year DOIs are only relatively recently becoming available. Faced with such uncertainty and a lack of clarity around what degree of protection is necessary, some guideline groups recommend booster vaccines every 3 years for all cats [10], whilst others recommend more frequent [9] or annual vaccination [31, 32] for cats at high risk of FCV and FeHV-1 infection, with the decision on how often to vaccinate being based on a risk-benefit assessment for individual cats. Whether the observed relatively frequent use of core vaccines in many cats in this population reflects a lack of guideline awareness, a decision between the veterinary surgeon and owner that most cats remain at high risk of FCV and FeHV-1, or whether the complex and sometimes conflicting language of guidelines is producing an inertia to change, will need to be determined.

The use of non-core vaccines was variable in this population. In dogs, *Leptospira* vaccines were generally used more frequently than core vaccines, consistent with its shorter DOI and contributing to the observed greater number of overall vaccines given to dogs. In cats, FeLV vaccines were used almost as frequently as core vaccines suggesting most veterinary surgeons consider most cats to be at risk of infection [9, 10, 33]; this contrasts data from 1999 suggesting that core vaccination was approximately twice as common as FeLV vaccination [34]. Rabies vaccine in some countries is a core vaccine [9, 10]. However, in GB, rabies vaccine is only mandated for animals coming to the UK from overseas and as such its use likely reflects animal importation and holiday travel. The preferential use of the *Bordetella* vaccine in dogs may suggest veterinary surgeons consider this upper respiratory tract pathogen a greater threat in the canine population, consistent with published data suggesting risk may be more widespread in dogs [35, 36] and more restricted in cats to large colonies [37, 38].

Guidelines and Summaries of Product Characteristics generally agree on kitten and puppy primary vaccinations; core vaccines are recommended at approximately 9–12 weeks of age, coinciding with declining maternally derived antibodies. Some recommend additional earlier and later (16 weeks-of-age) vaccines for some animals based on risk [10]. In the population studied here, we see clear peaks of canine vaccination around 12 weeks of age; the smaller amount of vaccination of younger puppies possibly reflecting animals having already received their first vaccine when acquired. In kittens, we did see evidence for these 9 and 12 week peaks in vaccination. In both species, there was little evidence to suggest later use of core vaccines at 16 weeks-of-age. A ‘booster’ vaccine is recommended for all kittens and puppies approximately 12 months after primary vaccination. Perhaps inevitably the timing of this vaccination was relatively dispersed in this population. However, of note, over one quarter of cats and dogs that had received at least one core vaccine in the first 6 months of life had no recorded booster vaccine suggesting a potential gap in immunity.

One of the big challenges for defining best vaccine protocols for cats and dogs has been an inability to carry out large post-authorisation studies on efficacy. As electronic health data becomes more available and more nuanced to include information on clinical outcome not just treatment, it is hoped that such trials will become more achievable. Such studies, when compiled with other pre-authorisation data and data already captured on adverse events [34], would help veterinary surgeons make the best informed risk-based decisions with their owners, on most appropriate vaccination schedules for individual pets.

Using health records from a sentinel network for research inevitably has limitations. Practices contributing data to SAVSNET are recruited on convenience so cannot necessarily be considered to be representative of the wider UK population. Individual NUTS1 regions are likely to have varied proportional coverage compared to total populations such that overall findings may be biased towards behaviours in regions with the greatest coverage (Supplementary Table 2). The Study Population can only include animals that attend participating veterinary practices and may miss individual vaccines that are either not recorded, or that are given at other practices such as vaccine clinics, or early-life vaccines given before the owner acquires the animal. Recruitment to the Study Population over almost 2 years may also bias the sample towards animals that visit their veterinary practice more frequently. Since we excluded animals born before their health records could be electronically recorded by their practice, the resulting Study Population was younger than the Total Population. The major limitation of the Full Antigen History dataset is that it excludes those animals lacking a complete record of the vaccine antigens used. Whilst we believe the robust protocol for data cleaning employed in this study minimises these limitations it does reduce the amount of available data. As the length of time EHRs are used by practices increases in relation to patient lifespans, some of these limitations will be gradually reduced. As well as publications, SAVSNET also uses these data to benchmark participating practices and it is it hoped this will lead to an improvement in the quality of vaccine antigen recording by participating practices.

In conclusion, the World Small Animal Veterinary Association vaccine guidelines include a call to vaccinate more animals [10]. Here for the first time, we quantify the level of vaccine uptake in a large sentinel population of UK practices, providing a benchmark against which trends in vaccination can be monitored. The risk factors we report can inform targeted health messages to reduce vaccine uptake variability; key targets include variation between practices and regions, those animals not receiving the first annual booster and potential opportunities to reduce vaccination frequency in those adult cats considered at low risk of infection.

## Supplementary material

For supplementary material accompanying this paper visit http://doi.org/10.1017/S0950268818000754. click here to view supplementary material

## References

[1] Briggs DJ (2012) The role of vaccination in rabies prevention. Current Opinions in Virology 2, 309–314. 10.1016/j.coviro.2012.03.007 22503445

[2] Gaskell RM, Dawson S and Radford AD (2006) Duration of immunity (DOI)—the regulatory issues. Veterinary Microbiology 117, 80–85. 16723198 10.1016/j.vetmic.2006.04.014

[3] Hartmann K, (2015) Feline injection-site sarcoma: ABCD guidelines on prevention and management. Journal of Feline Medicine and Surgery 17, 606–13. 26101312 10.1177/1098612X15588451 PMC11148925

[4] Scott FW and Geissinger CM (1999) Long-term immunity in cats vaccinated with an inactivated trivalent vaccine. American Journal of Veterinary Research 60, 652–8. 10328440

[5] Lakshmanan N, (2006) Three-year rabies duration of immunity in dogs following vaccination with a core combination vaccine against canine distemper virus, canine adenovirus type-1, canine parvovirus, and rabies virus. Veterinary Therapeutics 7, 223–31. 17039445

[6] Jas D, (2015) Three-year duration of immunity for feline herpesvirus and calicivirus evaluated in a controlled vaccination-challenge laboratory trial. Veterinary Microbiology 177, 123–31. 25824128 10.1016/j.vetmic.2015.03.009

[7] Day MJ, (2016) Vaccination Guidelines Group (VGG) of the World Small Animal Veterinary Association (WSAVA). WSAVA Guidelines for the vaccination of dogs and cats. Journal of Small Animal Practice 57, E1–E45. 26780857 10.1111/jsap.2_12431 PMC7166872

[8] Hosie MJ, (2015) Matrix vaccination guidelines. Journal of Feline Medicine and Surgery 17, 583–587. 26101309 10.1177/1098612X15590732 PMC11148924

[9] Richards JR, (2006) The 2006 American Association of Feline Practitioners Feline Vaccine Advisory Panel report. Journal of the American Veterinary Medical Association 229, 1405–41. 17078805 10.2460/javma.229.9.1405

[10] Day MJ, (2016) WSAVA Guidelines for the vaccination of dogs and cats. Journal of Small Animal Practice 57, E1–E45. 26780857 10.1111/jsap.2_12431 PMC7166872

[11] Asher L, (2011) Estimation of the number and demographics of companion dogs in the UK. BMC Veterinary Research 7, 74. 22112367 10.1186/1746-6148-7-74 PMC3305510

[12] Murray JK and Gruffydd-Jones TJ (2012) Proportion of pet cats registered with a veterinary practice and factors influencing registration in the UK. Veterinary Journal 192, 461–466. 21963659 10.1016/j.tvjl.2011.08.035

[13] Sánchez-Vizcaíno F, (2015) Small animal disease surveillance. Veterinary Record 177, 591–594. 26667432 10.1136/vr.h6174

[14] Sánchez-Vizcaíno F, (2017) Demographics of dogs, cats, and rabbits attending veterinary practices in Great Britain as recorded in their electronic health records. BMC Veterinary Research 13, 218. 28693574 10.1186/s12917-017-1138-9 PMC5504643

[15] Lesnoff M, Lancelot R (2012) Analysis of overdispersed data. Available at http://cran.r-project.org/package=aod.

[16] Team RC (2015) R: language and environment for statistical computing. Available at http://www.r-project.org/.

[17] Robinson NJ, (2016) Investigating preventive-medicine consultations in first-opinion small-animal practice in the United Kingdom using direct observation. Preventive Veterinary Medicine 124, 69–77. 26775818 10.1016/j.prevetmed.2015.12.010

[18] The People's Dispensary for Sick Animals (2016) PDSA Animal Wellbeing (PAW) Report: the state of our pet nation. Available at https://www.pdsa.org.uk/get-involved/our-current-campaigns/pdsa-animal-wellbeing-report.

[19] Jain A, (2017) Lower vaccine uptake amongst older individuals living alone: A systematic review and meta-analysis of social determinants of vaccine uptake. Vaccine 35, 2315–2328. 28343775 10.1016/j.vaccine.2017.03.013

[20] Hungerford D, (2016) Effect of socioeconomic deprivation on uptake of measles, mumps and rubella vaccination in Liverpool, UK over 16 years: a longitudinal ecological study. Epidemiology and Infection. 144, 1201–11. 26542197 10.1017/S0950268815002599

[21] Habacher G, Gruffydd-Jones T and Murray J (2010) Use of a web-based questionnaire to explore cat owners’ attitudes towards vaccination in cats. Veterinary Record 167, 122–7. 20656990 10.1136/vr.b4857

[22] Schultz RD (2006) Duration of immunity for canine and feline vaccines: A review. Veterinary Microbiology 117, 75–79. 16707236 10.1016/j.vetmic.2006.04.013

[23] Day MJ, Horzinek MC and Schultz RD (2007) Vaccination Guidelines Group (VGG) of the World Small Animal Veterinary Association (WSAVA). Guidelines for the vaccination of dogs and cats. Compiled by the Vaccination Guidelines Group (VGG) of the World Small Animal Veterinary Association (WSAVA). Journal of Small Animal Practice 48, 528–41. 17803726 10.1111/j.1748-5827.2007.00462.x PMC7167131

[24] Abdelmagid OY, (2004) Evaluation of the efficacy and duration of immunity of a canine combination vaccine against virulent parvovirus, infectious canine hepatitis virus, and distemper virus experimental challenges. Veterinary Therapeutics 5, 173–86. 15578450

[25] Gore TC, (2005) Three-year duration of immunity in dogs following vaccination against canine adenovirus type-1, canine parvovirus, and canine distemper virus. Veterinary Therapeutics 6, 5–14. 15906266

[26] Hou J, (2016) European molecular epidemiology and strain diversity of feline calicivirus. Veterinary Record 178, 114–5. 26811440 10.1136/vr.103446 PMC4752659

[27] Porter CJ, (2008) Comparison of the ability of feline calicivirus (FCV) vaccines to neutralise a panel of current UK FCV isolates. Journal of Feline Medicine and Surgery 10, 32–40. 17720588 10.1016/j.jfms.2007.06.011 PMC10911152

[28] Addie D, (2008) Ability of antibodies to two new caliciviral vaccine strains to neutralise feline calicivirus isolates from the UK. Veterinary Record 163, 355–7. 18806279 10.1136/vr.163.12.355

[29] Afonso MM, (2017) A multi-national European cross-sectional study of feline calicivirus epidemiology, diversity and vaccine cross-reactivity. Vaccine 35, 2753–2760. 28389099 10.1016/j.vaccine.2017.03.030

[30] Gaskell R, (2007) Feline herpesvirus. Veterinary Research 38, 337–54. 17296160 10.1051/vetres:2006063

[31] Radford AD, (2009) Feline calicivirus infection: ABCD guidelines on prevention and management. Journal of Feline Medicine and Surgery 11, 556–564. 19481035 10.1016/j.jfms.2009.05.004 PMC11132273

[32] Thiry E, (2009) Feline herpesvirus infection: ABCD guidelines on prevention and management. Journal of Feline Medicine and Surgery 11, 547–555. 19481034 10.1016/j.jfms.2009.05.003 PMC7129359

[33] Lutz H, (2009) Feline leukaemia. ABCD guidelines on prevention and management. Journal of Feline Medicine and Surgery 11, 565–74. 19481036 10.1016/j.jfms.2009.05.005 PMC7172531

[34] Gaskell RM, (2002) Veterinary Products Committee working group report on feline and canine vaccination. Veterinary Record 150, 126–34. 11871665

[35] Mochizuki M, (2008) Etiologic study of upper respiratory infections of household dogs. Journal of Veterinary Medical Sciences 70, 563–9. 10.1292/jvms.70.563 18628596

[36] Decaro N, (2016) Molecular surveillance of traditional and emerging pathogens associated with canine infectious respiratory disease. Veterinary Microbiology 192, 21–25. 27527760 10.1016/j.vetmic.2016.06.009 PMC7131703

[37] Helps CR, (2005) Factors associated with upper respiratory tract disease caused by feline herpesvirus, feline calicivirus, Chlamydophila felis and Bordetella bronchiseptica in cats: experience from 218 European catteries. Veterinary Record 156, 669–73. 15908495 10.1136/vr.156.21.669

[38] Binns SH, (1999) Prevalence and risk factors for feline Bordetella bronchiseptica infection. Veterinary Record 144, 575–80. 10378288 10.1136/vr.144.21.575

