# Supplementary material for: Use of vaccines and factors associated with their uptake variability in dogs, cats and rabbits attending a large sentinel network of veterinary practices across Great Britain
Source: Epidemiol Infect. 2018 Apr 11;146(7):895–903. doi: 10.1017/S0950268818000754 (PMC5960348; doi:10.1017/S0950268818000754)
Supplement: Supplementary file 1 [file S0950268818000754sup001.docx]

*Epidemiology and Infection*

Use of vaccines and uptake inequality in dogs, cats and rabbits attending veterinary practices measured by electronic health records.

F SÁNCHEZ-VIZCAÍNO, A MUNIESA, DA SINGLETON, PH JONES, PJ NOBLE, RM GASKELL, S DAWSON AND AD RADFORD.

**Supplementary material**

**Supplementary Table S1**

Shown are the results of each likelihood ratio test performed to evaluate the statistical significance of each random effect versus the other (i.e. “practice” Vs. “premise”) and versus both random effects included (“practice-premise”) on each dataset used in the study for fitting mixed effects models modelling the probability of being recorded as vaccinated in dogs, cats and rabbits attending a network of veterinary practices across Great Britain. In each comparison, the random effect that improved the fit of the model is highlighted in bold and with an asterisk (*). DF = degrees of freedom.

| Dataset analysed | Species | Country | Random effects tested | Likelihood ratio*χ*^2^ statistic | Difference in DF | *P* value |
| --- | --- | --- | --- | --- | --- | --- |
| Dogs with a recorded date of birth | Dog | GB | Practice *VS.* **Practice-Premises*** | 72.8 | 1 | <0.001 |
|  |  |  | Premises *VS.* **Practice-Premises*** | 86.5 | 1 | <0.001 |
| Cats with a recorded date of birth | Cat | GB | Practice *VS.* **Practice-Premises*** | 94.2 | 1 | <0.001 |
|  |  |  | Premises *VS.* **Practice-Premises*** | 31.4 | 1 | <0.001 |
| Rabbits with a recorded date of birth | Rabbit | GB | Practice *VS.* **Practice-Premises*** | 7.7 | 1 | 0.006 |
|  |  |  | Premises *VS.* **Practice-Premises*** | 30.0 | 1 | <0.001 |
| Dogs with a linked English IMD score | Dog | England | Practice *VS.* **Practice-Premises*** | 64.1 | 1 | <0.001 |
|  |  |  | Premises *VS.* **Practice-Premises*** | 78.4 | 1 | <0.001 |
| Cats with a linked English IMD score | Cat | England | Practice *VS.* **Practice-Premises*** | 52.8 | 1 | <0.001 |
|  |  |  | Premises *VS.* **Practice-Premises*** | 20.1 | 1 | <0.001 |
| Rabbits with a linked English IMD score | Rabbit | England | Practice *VS.* **Practice-Premises*** | 6.9 | 1 | 0.008 |
|  |  |  | Premises *VS.* **Practice-Premises*** | 24.7 | 1 | <0.001 |
| Dogs with a linked Scottish IMD score | Dog | Scotland | Practice *VS.* **Practice-Premises*** | 6.9 | 1 | 0.009 |
|  |  |  | Premises *VS.* **Practice-Premises*** | 18.4 | 1 | <0.001 |
| Cats with a linked Scottish IMD score | Cat | Scotland | Practice *VS.* **Premises*** | 29.0 | 0 | <0.001 |
|  |  |  | Premises *VS.* Practice-Premises | 0.7 | 1 | 0.4 |
| Rabbits with a linked Scottish IMD score | Rabbit | Scotland | Practice *VS.* **Premises*** | 2.0 | 0 | <0.001 |
|  |  |  | Premises *VS.* Practice-Premises | 0.1 | 1 | 0.8 |
| Dogs with a linked Welsh IMD score | Dog | Wales | Premises *VS.* **Practice*** | 8.2 | 0 | <0.001 |
|  |  |  | Practice *VS.* Practice-Premises | 0 | 1 | 1 |
| Cats with a linked Welsh IMD score | Cat | Wales | Practice *VS.* **Premises*** | 13.7 | 1 | <0.001 |
|  |  |  | Premises *VS.* Practice-Premises | 0 | 1 | 1 |
| Rabbits with a linked Welsh IMD score | Rabbit | Wales | Premises *VS.* **Practice*** | 1.6 | 0 | <0.001 |
|  |  |  | Practise *VS.* Practice-Premises | 0 | 1 | 1 |

**Supplementary Table S2.** Number of animals included in the study population and their recorded vaccination levels in each region of Great Britain, stratified by species.

| Region | Species | Number of animals | Percentage of animals with at least one recorded vaccine (95% CI) |
| --- | --- | --- | --- |
| East Midlands | Total | 6,833 | 77.5 (76.5-78.5) |
|  | Dog | 4,764 | 81.1 (80.0-82.2) |
|  | Cat | 1,903 | 71.4 (69.3-73.4) |
|  | Rabbit | 166 | 43.4 (36.1-51.0) |
| East of England | Total | 6,634 | 82.3 (81.4-83.2) |
|  | Dog | 4,167 | 86.1 (85.0-87.1) |
|  | Cat | 2,248 | 77.9 (76.1-79.6) |
|  | Rabbit | 219 | 54.3 (47.7-60.8) |
| London | Total | 3,244 | 76.7 (75.3-78.2) |
|  | Dog | 1,729 | 77.6 (75.5-79.5) |
|  | Cat | 1,444 | 76.6 (74.3-78.7) |
|  | Rabbit | 71 | 57.7 (46.1-68.5) |
| North East | Total | 8,083 | 78.1 (77.1-78.9) |
|  | Dog | 5,876 | 81.8 (80.8-82.8) |
|  | Cat | 1,911 | 72.2 (70.1-74.1) |
|  | Rabbit | 296 | 41.5 (36.1-47.2) |
| North West | Total | 17,356 | 79.6 (79.0-80.2) |
|  | Dog | 11,258 | 84.2 (83.5-84.9) |
|  | Cat | 5,337 | 76.2 (75.1-77.4) |
|  | Rabbit | 761 | 34.7 (31.4-38.1) |
| Scotland | Total | 9,256 | 74.7 (73.8-75.6) |
|  | Dog | 6,561 | 75.4 (74.3-76.4) |
|  | Cat | 2,436 | 76.6 (74.9-78.3) |
|  | Rabbit | 259 | 40.1 (34.4-46.2) |
| South East | Total | 20,024 | 81.4 (80.9-82.0) |
|  | Dog | 12,398 | 84.6 (84.0-85.2) |
|  | Cat | 6,981 | 77.2 (76.2-78.2) |
|  | Rabbit | 645 | 65.4 (61.7-69.0) |
| South West | Total | 9,256 | 77.2 (76.3-78.0) |
|  | Dog | 6,423 | 81.4 (80.4-82.3) |
|  | Cat | 2,588 | 69.0 (67.2-70.7) |
|  | Rabbit | 245 | 52.6 (46.4-58.8) |
| Wales | Total | 8,384 | 70.5 (69.6-71.5) |
|  | Dog | 5,814 | 76.8 (75.7-77.9) |
|  | Cat | 2,354 | 56.4 (54.4-58.4) |
|  | Rabbit | 216 | 54.2 (47.5-60.7) |
| West Midlands | Total | 9,372 | 74.9 (74.1-75.8) |
|  | Dog | 5,950 | 78.0 (77.0-79.1) |
|  | Cat | 3,018 | 71.4 (69.7-73.0) |
|  | Rabbit | 404 | 55.9 (51.1-60.7) |
| Yorkshire and The Humber | Total | 13,290 | 81.9 (81.2-82.5) |
|  | Dog | 9,662 | 85.7 (85.0-86.4) |
|  | Cat | 3,267 | 74.8 (73.3-76.3) |
|  | Rabbit | 361 | 43.23 (38.2-48.4) |

**Supplementary Table S3**

Number of animals included in the study population stratified by species, British country and Index of Multiple Deprivation (IMD). IMD category 1 indicates the least deprived areas and category 5 the most deprived. In each species, the percentages shown in brackets are calculated column wise and at country level.

| Country | IMD | Number of dogs  (percentage) | Number of cats (percentage) | Number of rabbits (percentage) |
| --- | --- | --- | --- | --- |
| England | 1 | 16,726 (26.88) | 7,954 (27.73) | 975 (30.86) |
|  | 2 | 16,935 (27.22) | 6,963 (24.27) | 791 (25.04) |
|  | 3 | 14,214 (22.84) | 6,503 (22.67) | 686 (21.72) |
|  | 4 | 8,522 (13.70) | 4,531 (15.79) | 428 (13.55) |
|  | 5 | 5,821 (9.36) | 2,737 (9.54) | 279 (8.83) |
| Scotland | 1 | 1,620 (24.69) | 662 (27.19) | 89 (34.50) |
|  | 2 | 1,939 (29.56) | 732 (30.06) | 64 (24.81) |
|  | 3 | 1,557 (23.73) | 525 (21.56) | 63 (24.42) |
|  | 4 | 949 (14.47) | 323 (13.26) | 23 (8.91) |
|  | 5 | 495 (7.55) | 193 (7.93) | 19 (7.36) |
| Wales | 1 | 193 (3.54) | 78 (3.55) | 6 (2.94) |
|  | 2 | 1,617 (29.65) | 592 (26.96) | 60 (29.41) |
|  | 3 | 1,556 (28.54) | 742 (33.79) | 74 (36.27) |
|  | 4 | 1,039 (19.05) | 414 (18.85) | 30 (14.71) |
|  | 5 | 1,048 (19.22) | 370 (16.85) | 34 (16.67) |
